# Fix Your Membrane Receptor Imaging: Actin Cytoskeleton and CD4 Membrane Organization Disruption by Chemical Fixation

**DOI:** 10.3389/fimmu.2019.00675

**Published:** 2019-04-05

**Authors:** Pedro M. Pereira, David Albrecht, Siân Culley, Caron Jacobs, Mark Marsh, Jason Mercer, Ricardo Henriques

**Affiliations:** ^1^MRC-Laboratory for Molecular Cell Biology, University College London, London, United Kingdom; ^2^The Francis Crick Institute, London, United Kingdom; ^3^Institute for the Physics of Living Systems, University College London, London, United Kingdom; ^4^Department of Cell and Developmental Biology, University College London, London, United Kingdom

**Keywords:** super-resolution imaging, CD4, actin cortex, fixation, artefact analysis

## Abstract

Single-molecule localization microscopy (SMLM) techniques allow near molecular scale resolution (~ 20 nm) as well as precise and robust analysis of protein organization at different scales. SMLM hardware, analytics and probes have been the focus of a variety of studies and are now commonly used in laboratories across the world. Protocol reliability and artifact identification are increasingly seen as important aspects of super-resolution microscopy. The reliability of these approaches thus requires in-depth evaluation so that biological findings are based on solid foundations. Here we explore how different fixation approaches that disrupt or preserve the actin cytoskeleton affect membrane protein organization. Using CD4 as a model, we show that fixation-mediated disruption of the actin cytoskeleton correlates with changes in CD4 membrane organization. We highlight how these artifacts are easy to overlook and how careful sample preparation is essential for extracting meaningful results from super-resolution microscopy.

## Introduction

Super-resolution microscopy is a fundamental tool for exploring and understanding nanoscale biological assemblies. Single-molecule localization microscopy (SMLM) techniques in particular, such as photoactivated localization microscopy (PALM) (1) and stochastic optical reconstruction microscopy (STORM) (2), are the optical imaging gold standards to study membrane protein organization (3). SMLM techniques provide high spatial resolution (~ 20 nm) and allow for statistical, nonbiased analysis of membrane protein nanoscale organizations (1, 2, 4, 5). Thereby, super-resolution microscopy has provided new views on the organization of membrane receptors, from immune sensing to pathogen engagement (6). The organization of receptors into micro- and nanoclusters at the plasma membrane is a common feature and an important regulatory mechanism for cell signaling and activation (7–12). Thus, analyzing the nanoscale level organization of these molecules is critical to understand basic regulation of cellular signaling but also to understand the function of these proteins in disease. For example, CD4 plays an important role in immune cell activation through its ability to enhance T-cell receptor (TCR)-mediated signaling by binding to the antigen-presenting major histocompatibility complex II (MHCII) (13). Besides its importance in immune signaling, CD4 is also the primary cellular receptor for human immunodeficiency viruses (HIV) (13, 14). The importance of super-resolution in the study of membrane receptor organization and function cannot be overstated. A recent example is the characterization of the spatiotemporal dynamics and stoichiometry of the interactions between CD4 (and co-receptors) and HIV-1 in the context of viral entry, impossible to achieve without molecular imaging approaches (14).

A key component of membrane organization is the actin cytoskeleton (15, 16). The actin cortex underlies the plasma membrane and interacts with both lipids and membrane proteins, functioning as a dynamic scaffold providing support and force for the continuous remodeling of membrane receptor organization (17–19). It is not surprising that the actin cytoskeleton has been the subject of a considerable number of studies in a variety of biological settings, from viral engagement to axon organization using (super-resolution) microscopy (17, 20, 21).

The increased resolution and detailed analytic information provided by SMLM requires rigorous scrutiny of collected data (22–24). The succession of steps from the native organization of a receptor in the plasma membrane to the final super-resolution image can be significantly influenced by artifacts, particularly if imaging requires chemical fixation (22–24). Ideally, chemical fixation preserves the macroscopic structure of the sample as well as the native nanoscale organization of target proteins. However, true preservation at the subcellular level is not trivial, as known from electron microscopy studies (25, 26). Furthermore, chemical fixation does not immediately immobilize membrane-associated proteins (27). Thus, given the increase in resolution afforded by super-resolution microscopy, the effect of fixation has been the focus of several recent studies (22–24). Importantly, there are multiple chemical fixation methods, differing by the fixative used (e.g., paraformaldehyde, glutaraldehyde, glyoxal or methanol), the buffer composition (e.g., phosphate buffered saline, cytoskeleton stabilizing buffer or PIPES-EGTA-magnesium buffer), and physical conditions (temperature and duration) (22–24, 28–30). There is, at this stage, no standardized sample preparation protocol to study membrane protein organization. Moreover, to the best of our knowledge, there is no correlative study to understand how, in the same cells, fixation-induced changes in the actin cytoskeleton may affect membrane protein organization.

**Figure 1:**
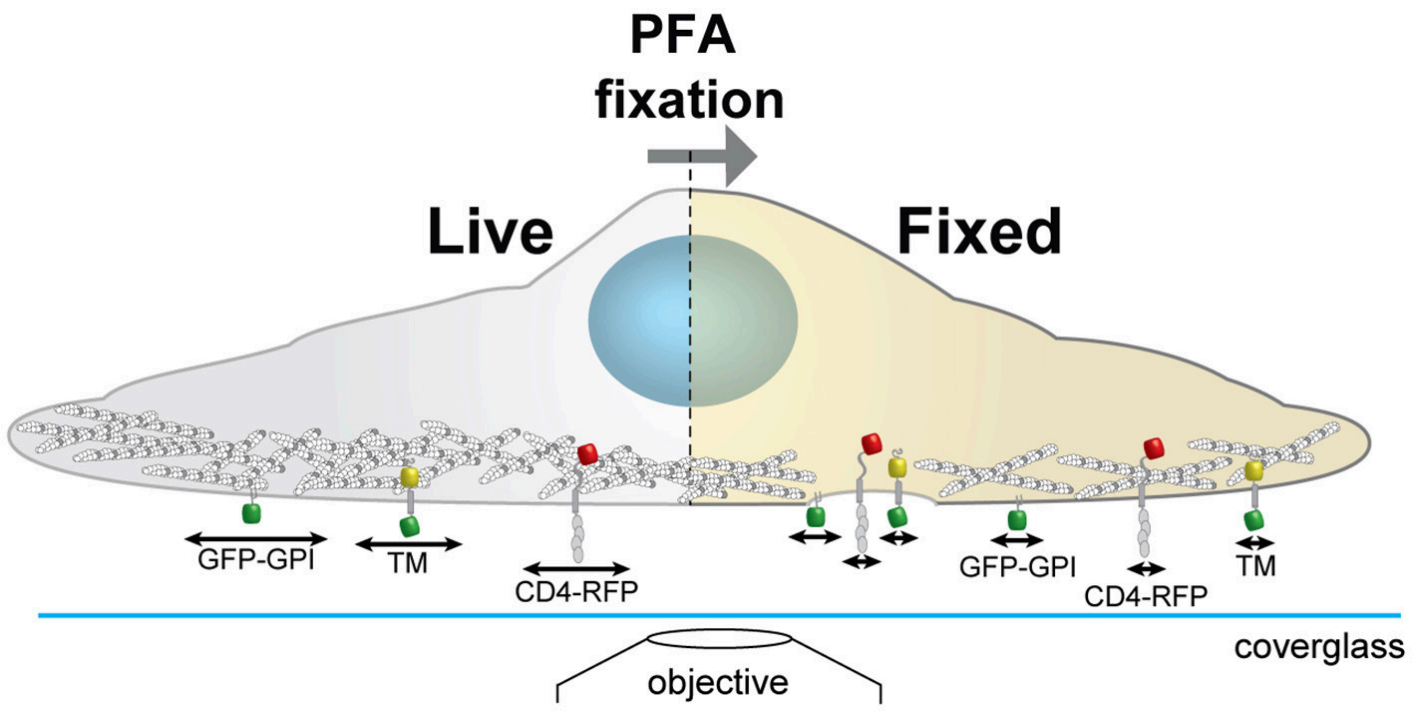
Schematics of the experimental workflow to correlate actin morphology with CD4 membrane organization. We analyse on the same cells how the actin cytoskeleton morphology changes with different chemical fixation protocols and how this correlates with the membrane organization and mobility of CD4. Cortical actin (white and gray circles); arrows represent protein mobility; GPI anchored GFP (GFP-GPI); artificial transmembrane protein with cytosolic and extracellular domains (mHoneydew and YFP, respectively - TM); CD4 fused to TagRFP-T (CD4-RFP).

Here, we analyze how the morphology of the actin cytoskeleton changes with different chemical fixation protocols and how these changes correlate with the membrane organization of the membrane receptor CD4 (Figure 1). We show that conditions that have detrimental effects on cytoskeleton organization correlate with changes in the membrane organization of CD4. We suggest that careful sample preparation and handling during all steps leading to the final image is essential for all scientists.

### Suboptimal Fixation Protocols Affect the Actin Cytoskeleton and CD4 Membrane Organization Differently

**Figure 2:**
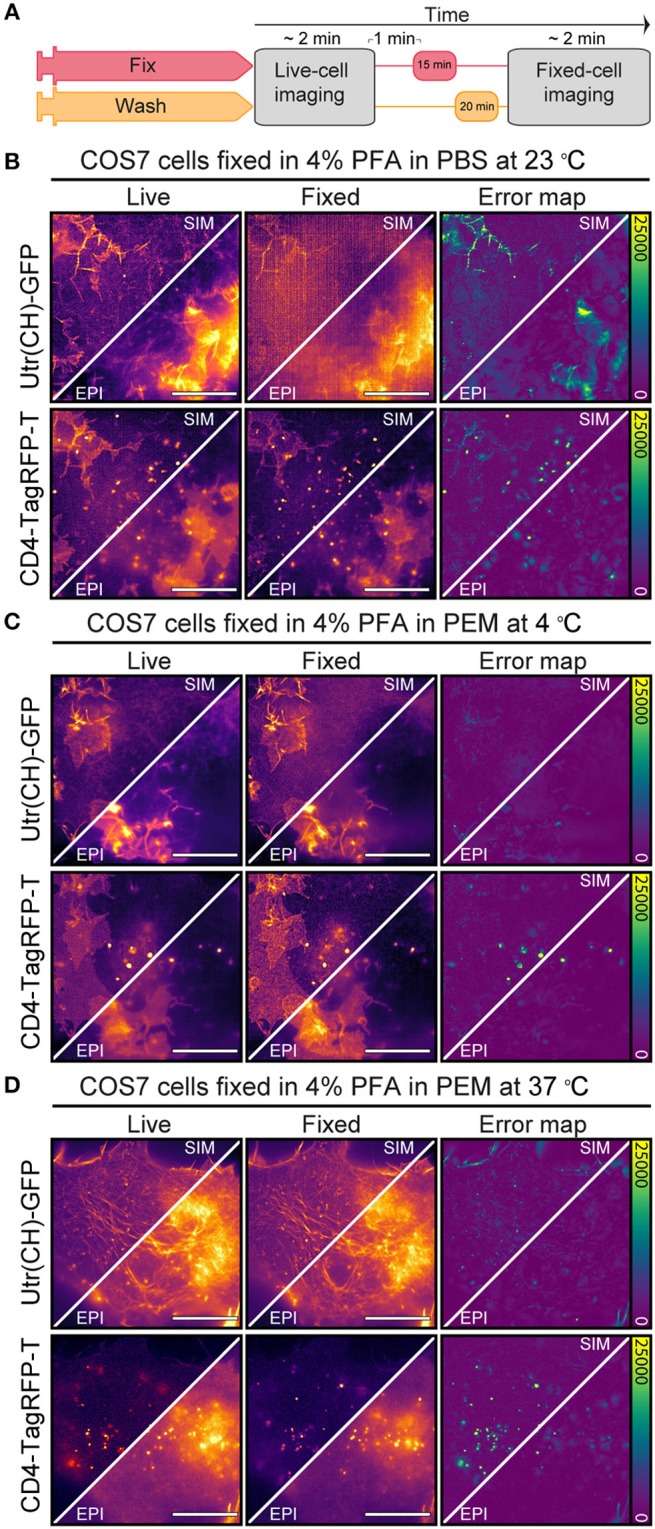
Effect of suboptimal fixation conditions on actin and CD4 organization. **(A)** NanoJ-Fluidics protocol to perform the live-to-fixed cell correlation under different suboptimal fixation conditions. **(B)** Epifluorescence and SIM imaging of COS7 cell expressing Utr(CH)-GFP and CD4-TagRFP-T live (Live) and fixed (Fixed) with 4% PFA in PBS at 23°C and corresponding NanoJ-SQUIRREL error maps (Error map). **(C)** same as in **(B)** but fixation was performed with 4% PFA in PEM at 4°C. **(D)** same as in **(B)** but fixation was performed with 4% PFA in PEM at 37°C. Scale bars are 10 μm.

To understand the effect of suboptimal actin fixation protocols on CD4 membrane organization we correlated live-cell and fixed-cell actin and CD4 organization using NanoJ-Fluidics (31) (Figure 2A) and Structured Illumination Microscopy (SIM) (32).We imaged actin in live COS7 cells with an utrophin domain (UtrCH-GFP) (33) probe and CD4 tagged with TagRFP-T. We performed chemical fixation using three different chemical fixation protocols, 4% paraformaldehyde (PFA) in PBS at 23°C, 4% PFA in PEM (23) at 4°C or at 37°C (Figures 2B–D). Subsequently, using NanoJ-SQUIRREL (22), to compare the live-cell vs. fixed-cell organization of actin and CD4, we were able to identify the effects of the suboptimal (4% PFA in PBS at 23°C and 4% PFA in PEM at 4°C) fixation protocols on these targets and compare with the optimal protocol (37°C 4% PFA in PEM) (23). As expected (23, 24), using PBS we observed a loss of protrusive actin-based structures and actin stress fibers appear to be disassembled or disrupted (Figure 2B). The fixation resulted in an almost indiscernible actin cytoskeleton, which translates to a NanoJ-SQUIRREL error map exhibiting strong artifacts (Figure 2B). Using PEM buffer, more suited for actin preservation (23), but at a suboptimal temperature (4°C), we see less of the aforementioned defects on the actin organization (Figure 2C). Pre-warming the PFA-containing PEM buffer to 37°C yielded a similar difference between live and fixed sample as measured by NanoJ-SQUIRREL (Figure 2D). Regardless of the fixation approach we did not see an effect on CD4 membrane organization, quantified on the error maps where most of the differences are due to vesicle motion during fixation (Figures 2B–D).

### The Fixation Protocol Influences CD4 Cluster Size and Cluster Density at the Cell Surface

**Figure 3:**
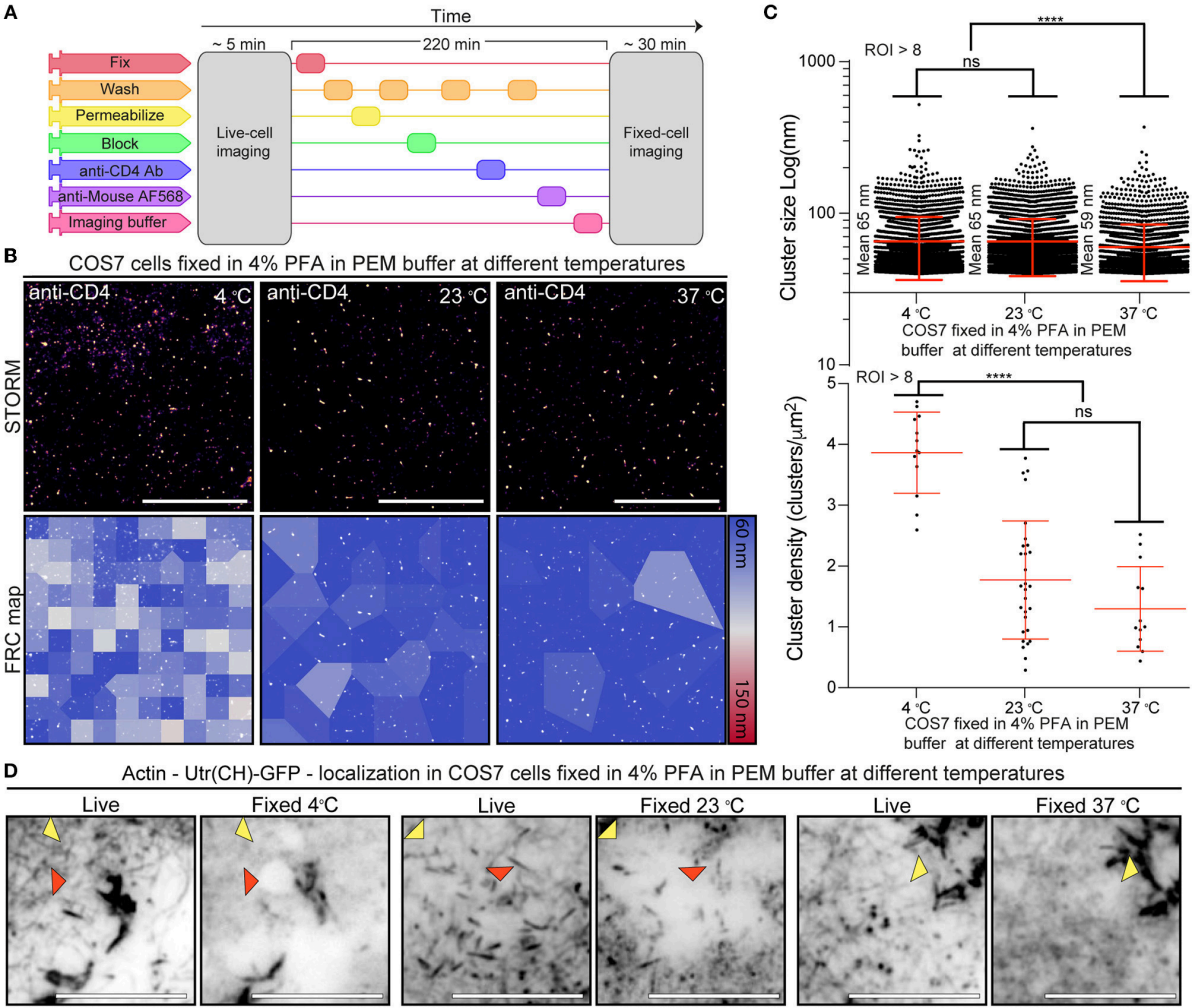
Correlation between CD4 membrane organization and actin structure fidelity upon fixation **(A)** NanoJ-Fluidics protocol to perform live-to-fixed cell correlation under different fixation conditions. **(B)** CD4 STORM imaging after fixation in different conditions (Top) and FRC map of the same region (Bottom). Scale bars are 5 μm. **(C)** CD4 cluster size and cluster density under different fixation conditions. **(D)** Diffraction limited (TIRF) live-to-fixed cell correlation using different fixation conditions. Red arrowheads highlight areas where actin disappeared upon fixation. Yellow arrowheads highlight areas where there is a difference in actin organization due to fixation. Scale bars are 1 μm. ^****^p < 0.001.

To ascertain if CD4 membrane organization was correlated with fixation-mediated actin cytoskeleton disruption we repeated the live-to-fixed cell correlation using SMLM and PEM with different fixation temperatures. PEM is an ideal buffer for actin preservation (23), and the range of temperatures provide different fixation efficiencies, with decreasing efficiency from 37°C (ideal) to 23°C (intermediate) to 4°C (lowest efficiency). We took advantage of the versatile NanoJ-Fluidics (31) framework to correlate live and fixed cell imaging of COS7 cells (Figure 3A). As expected, regardless of the fixation strategy we obtain a fairly homogeneous distribution of CD4 on the surface of COS7 cells (Figure 3B) at an in-cell high-resolution [43–50 nm by FRC (22)]. To further explore the nature of the CD4 organization we used SR-Tesseler (4) to determine if the cluster sizes and cluster density of CD4 would change depending on the fixation approach (Figure 3C). Interestingly, despite the little changes observed by SIM (Figure 2), both CD4 cluster size and cluster density changed with the fixation approach. Whereas, the mean CD4 cluster size in ideal conditions (PEM buffer at 37°C) is 59 nm, reducing the temperature to 23 or 4°C is enough to change CD4 organization, increasing the mean cluster size to 65 nm (*p* < 0.001), albeit these differences are likely not biologically relevant (see section Discussion). The fixation conditions also influence the CD4 cluster density in COS7 cells, with densities of 1.3 clusters/μm^2^ at 37°C, 1.8 clusters/μm^2^ at 23°C, and 3.8 clusters/μm^2^ at 4°C.

### Fixation-Induced CD4 Reorganization Correlates With Actin Cytoskeleton Preservation

We posited that fixation-induced changes in CD4 organization could be related to disruption of the actin cytoskeleton (Figure 2). To determine if the actin cytoskeleton was affected we compared the actin organization in the cells pre- and post-fixation (Figure 3D). We observed a disruption of the actin cytoskeleton at 23 and 4°C when compared with fixation at 37°C (Figure 3D). Independent of the fixation condition the post-fixation actin organization is different from the live-cell actin organization (Figure 3D yellow arrowheads). With the decrease in fixation temperature there is a step-wise decrease in the fidelity of the fixed-cell actin structure in relation to the one observed in live-cells. At lower fixation temperatures, actin filaments disappear and there are gaps in the actin structure, possibly related to cell detachment from the substrate or actin cytoskeleton disruption (Figure 3D red arrowheads). These artifacts are less prevalent in cells fixed under conditions that preserve the actin cytoskeleton structure.

### CD4 Membrane Reorganization Is not Related to Fixation-Induced Cell Membrane Disruption

**Figure 4:**
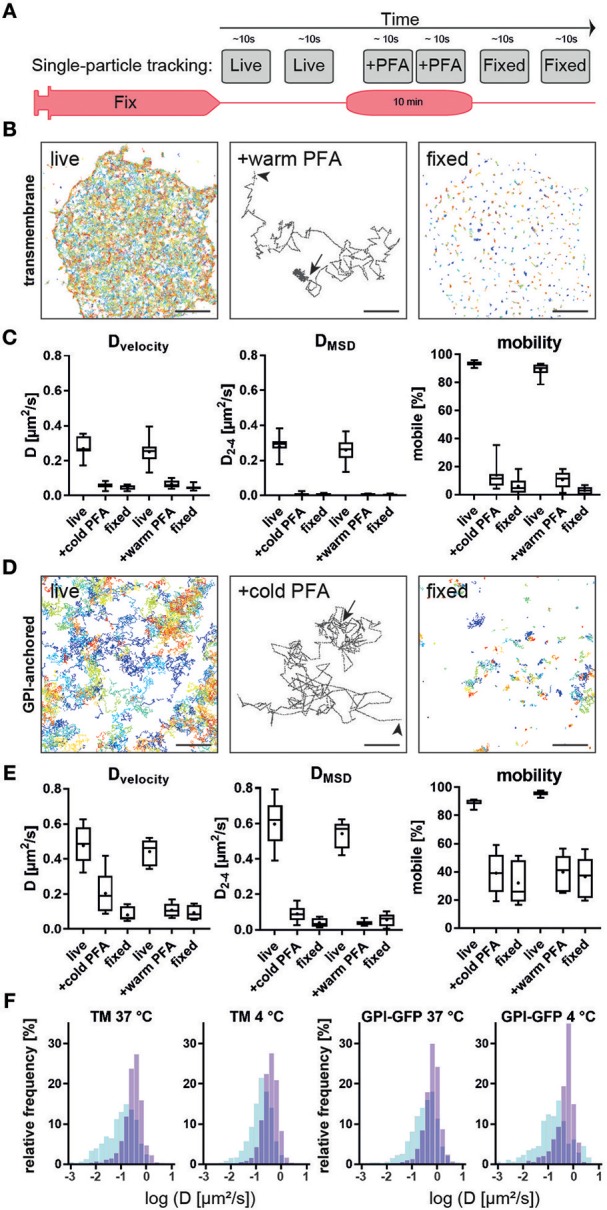
Single-particle tracking of membrane probes during live fixation. **(A)** Experimental workflow for live and fixed cell single-particle tracking. **(B)** Trajectories of a transmembrane probe with cytosolic and extracellular domains, tracked via fluorescently-labeled nanobodies on live cells (left). Middle panel show a typical trajectory with Brownian motion at the start (arrowhead) and immobilization upon addition of 4% PFA in PEM buffer (arrow). All transmembrane proteins appear immobilized in fixed cells. **(C)** Quantification of diffusion coefficient D based on velocity (left), mean-square displacement (middle) and percentage of mobile (D>0.05μm^2^/s) particles (right). No significant difference between chemical fixation at 4 or 37°C was observed. **(D)** Trajectories of GPI-anchored probe tracked via fluorescently labelled nanobodies on live cells (left). Middle panel show a typical trajectory with Brownian motion at the start (arrowhead) and reduced mobility upon addition of 4% PFA in PEM buffer (arrow). Some GPI-anchored proteins are immobilized in fixed cells while a fraction remains mobile. **(E)** Quantification of diffusion coefficient D based on velocity (left), mean-square displacement (middle) and percentage of mobile (D>0.05μm^2^/s) particles (right). **(F)** No significant difference between chemical fixation at 4 or 37°C was observed despite a trend toward faster fixation at warmer temperatures. Scale bars are 5 μm (left, right) and 500 nm (middle panels).

The difference in membrane receptor organization could be the result of the dependence of fixation efficiency on temperature. Employing our live-to-fix approach, we sought to determine how quickly the addition of PFA-containing PEM buffer immobilizes membrane associated proteins (Figure 4). An artificial transmembrane protein with a ~30 kDa cytosolic and extracellular domain (mHoneydew and YFP, respectively) was expressed in COS7 cells and individual proteins tracked with uPAINT (34), i.e., by adding low concentration (~20 pMol) of Atto647N labeled anti-GFP nanobodies (Chromotek) to the medium (Figure 4B, first panel). Diffusion coefficients based on particle velocity were 0.27 ±0.06 μm^2^/s (mean). Exchanging the cell culture medium with 37°C pre-warmed 4% PFA in PEM immediately reduced the diffusion speed of transmembrane proteins (Figure 4B, middle panel, arrow) and, after 10 min fixation, 97% of proteins were immobilized (*D* < 0.05 μm^2^/s) (Figure 4C). Addition of cold (4°C) 4% PFA in PEM had similar effects on measured diffusion coefficients and mobility (Figure 4C). Next, we tested if the same was true for a GPI-anchored protein that lacks any cytosolic domain that might interact directly with the cytoskeleton. GPI-anchored GFP was tracked via anti-GFP nanobodies. Addition of warm (37°C) or cold (4°C) 4% PFA in PEM buffer to live cells reduced the mobility of tracked individual particles without immobilizing them completely (Figure 4D, middle panel, arrow). In contrast to the transmembrane probe, only some particles were immobilized after fixation for 10 min. The reduction of diffusion coefficients as measured by velocity or mean square displacement (Figure 4E, left and middle panel) was not significantly different based on temperature. The mobile fraction was reduced to 36 and 32% (mean) after warm and cold fixation, respectively. Thus, changes in diffusive behavior were more dependent on the type of membrane protein tracked, rather than the fixation conditions (Figures 4C,E), which is in agreement with previous publications (27). However, even with only 4% PFA and without any cross-linking fixatives, we observed a rapid immobilization of transmembrane proteins that would prevent artificial clustering by subsequent antibody labeling approaches. Comparison of trajectories during the 10 s before and after addition of the chemical fixative showed an immediate shift of the histogram of diffusion coefficients determined via MSD toward lower values (Figure 4F). While there was no striking difference between pre-warmed and ice-cold fixative, we observed a trend toward a faster decrease in mobility at the higher temperature.

### Chemical Fixation Immediately Stops Cellular Motion

**Figure 5:**
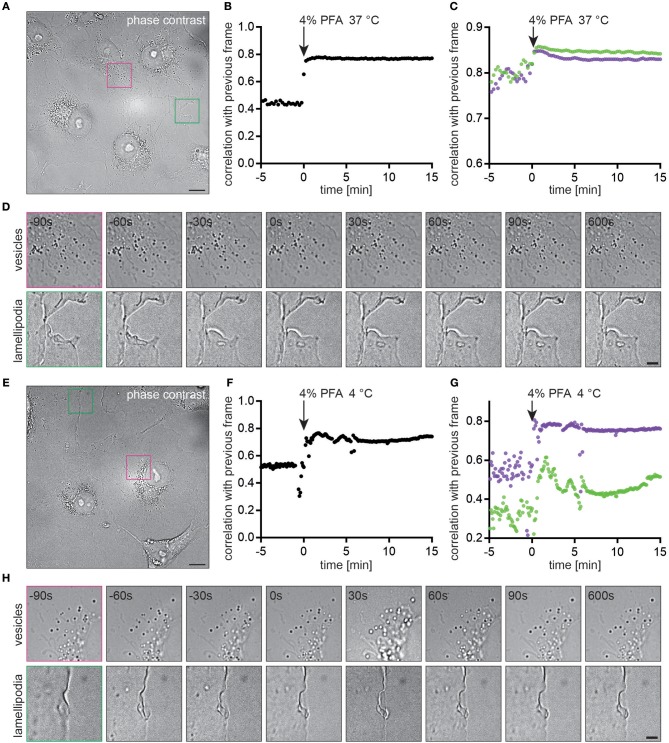
Cellular motion is immediately stopped by chemical fixation with PFA **(A)** Live COS7 cell imaged with phase contrast microscopy. **(B)** Image correlation between subsequent frames indicates an immediate stop of cellular processes at *t* = 0 min when the medium was replaced with warm 4% PFA. **(C)** Correlations for regions of interest with vesicles (purple) or lamellipodia (green). **(D)** Time-lapse of images before and after addition of the chemical fixative. Cellular features become static within 30 s. **(E)** Live COS7 cell imaged with phase contrast microscopy. **(F)** Image correlation between subsequent frames indicates an immediate stop of cellular processes at *t* = 0 min when the medium was replaced with cold 4% PFA. **(G)** Correlations for regions of interest with vesicles (purple) or lamellipodia (green). **(H)** Time-lapse of images before and after addtion of the chemical fixative. Cellular features become static within 30 s. Scale bars are 10 μm **(A,E)** and 3 μm **(D,H)**.

We determined how long cellular processes such as the motion of intracellular vesicles or lamellipodia persist during chemical fixation with PFA and whether this process was temperature dependent. Live COS7 cells were imaged in phase-contrast (Figure 5A). Upon exchange of the medium with 4% PFA pre-warmed to 37°C all cellular motion stopped immediately as determined by correlating images with the previous frame for the entire field of view (Figure 5B) or selected regions (Figures 5C,D and Supplementary Movies 1, 2). The plateaus in correlations pre- and post PFA addition correspond to cellular motion and noise during imaging. Chemical fixation with ice-cold 4% PFA inhibited cellular motion equally fast (Figures 5E–G). The increased fluctuations in correlations (Figures 5F,G) were caused by a shift in the focal plane, also observed in the image sequence (Figure 5 and Supplementary Movies 3, 4).

### Discussion

The super-resolution revolution in optical microscopy offers even inexperienced users up to 10-fold increased resolution on commercial systems that have become commonly available through imaging facilities. However, established sample preparation protocols that were previously acceptable may be inadequate for super-resolution microscopy, as the inaccuracies are no longer masked by the diffraction limit. While the importance of careful sample preparation is readily accepted, its assessment remains challenging. Neglecting to recognize this cost associated with increased resolution could render imaging results useless or worse might incorrectly inform researchers about a biological system. To demonstrate sample preparation inadequacies in imaging regimes, we took advantage of NanoJ-Fluidics (31) and NanoJ-SQUIRREL (22) to compare the pre- and post-fixation actin structures and CD4 cellular organization, in the same cells. We asked what would be the influence of chemical fixation using different imaging regimes with increasing resolution (TIRF, SIM and SMLM) by correlating pre- and post-fixation images. The actin cytoskeleton acts as a supporting scaffold that orchestrates the organization of the plasma membrane (35, 36). However, while actin filaments are strongly affected by chemical fixation conditions, the plasma membrane itself is affected to a lesser extent. Chemical fixation is usually fast and even a simple protocol can achieve structural preservation of the organization of transmembrane proteins in the plasma membrane. Despite the availability of chemical fixation protocols that preserve the actin cytoskeleton, the predominant approach for studying protein organization is fixation with 4% PFA in PBS. Our data suggests this is insufficient to produce reliable imaging data on receptor distributions for imaging modalities that break the diffraction limit. The chemical fixation protocol used was shown to play a crucial role on the introduction of artifacts. We applied SQUIRREL, a recently developed quality metric tool (22), to quantify how much cytoskeletal structures are distorted by chemical fixation at exemplary conditions. Our approach is widely applicable to determine the impact of any fixation protocol beyond those tested. Of course, a correlation between pre- and postfixation structures is required which, albeit greatly facilitated by NanoJ-Fluidics (31), is still a time-consuming quality control approach. However, in our opinion, the benefit of increased confidence in light microscopy data is worth the added effort. The increase in cluster size and density we observed could be due to: (1) disruption of the actin cytoskeleton organization that could affect to CD4 membrane organization via protein-protein interaction; (2) fixation-induced changes in membrane properties, which would cause artificial reorganization of membrane proteins; (3) a combination of both factors. Using super-resolution microscopy we could show that the changes in CD4 organization coincided with a disrupted actin cytoskeleton profile. The cluster size in optimal conditions suggests CD4 may be organized in dimers (as seen by the mean cluster size of ~60 nm), which is consistent with its suggested capacity to homo-dimerize, a process that may increase the avidity of its binding to MHCII (37). The differences observed between temperatures regarding CD4 cluster size are negligible (~6 nm) and likely related to the high number of data points skewing the statistical analysis. This is something the reader should always have in mind when analyzing statistical significance, as in this case the ~6 nm is significantly below the resolution our setup can provide and within the linker error introduced by using antibodies (~10 nm). The cluster density suggests a homogeneous distribution consistent with COS7 non-native CD4 expression. It is important to highlight that the considerable differences in cluster density are in a system where CD4 does not normally exist, hence lacking the regulatory machinery or native interactions that may normally regulate CD4 distribution. Presumably, the observed differences would be more striking in CD4-positive immune cells where CD4 is linked to p56/LCK (38). Interestingly, the degree of actin cytoskeleton disruption is consistent with the extent of the changes we observe in CD4 membrane organization. After chemical fixation at 4°C we observed almost complete disruption whereas at 23°C the cell displays a mixture of regions with disrupted and non-disrupted actin structures. This suggests that despite CD4 not existing in COS7 cells in native conditions, CD4 organization may be affected by the structure of the dense actin cortex (possibly through its cytoplasmic domain). Consequently, inadequate actin chemical fixation regimes can affect CD4 membrane organization and influence the biological information extracted from SMLM CD4 analysis. Challenging fields, such as the spatial distribution of immunomodulatory receptors require rigorous controls. For example, actin cytoskeleton dynamics affect clustering in immunological synapses (39, 40). Our approach could be employed to quantify the effects of actin perturbing drugs used on these cells.

We cannot exclude that membrane disruption and reorganization (such as membrane permeabilization or steep temperature mismatches between live-cell and fixation buffers, respectively) also plays a role in exacerbating the differences we observe. The importance of membrane composition and organization for surface protein distribution is well-known (41–43). Nonetheless, an indirect actin-related effect cannot be disregarded. The link between membrane composition and actin regulation is also recognized (44). For example, it is known that the pool of actin monomers is modulated by phosphoinositides (45, 46), or that alterations in the levels of cholesterol can change the membrane-cytoskeleton adhesion properties (47). However, our objective is to inform the reader on the possible outcomes that common sample preparation approaches (as multi-target IF or the use of intracellular epitopes, or different fixation temperatures) may have. If possible, cell membrane permeabilization and steep temperature changes should be avoided for their effect on the sample. Additionally, thermal drift affecting the optical system may reduce image quality or introduce artifacts.

**Figure 6:**
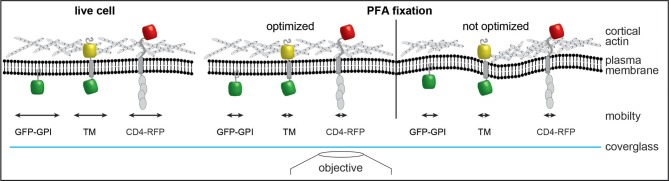
Model of changes induced by chemical fixation on membrane architecture. Optimized fixation with PFA preserves the cortical actin cytoskeleton structure in a state resembling live imaging and rapidly stops diffusion of transmembrane proteins. Suboptimal fixation conditions induce deformations of membrane and cytoskeleton and could thereby introduce artifacts. While the mobility of membrane probes is reduced similarly to optimized chemical fixation the overall organization could be altered due to interruptions of the cytoskeleton. GPI anchored GFP (GFP-GPI); artificial transmembrane protein with cytosolic and extracellular domains (mHoneydew and YFP, respectively - TM); CD4 fused to TagRFP-T (CD4-RFP).

These results are further supported by single-particle tracking experiments. Single-particle tracking of transmembrane proteins and a GPI-anchored protein showed that the size and orientation in the plasma membrane was more important than fixation conditions. GPI-anchored proteins that reside in the outer leaflet of the plasma membrane with only indirect interaction with the submembrane cytoskeleton (48) remain largely mobile in ideal actin-preserving conditions. Any distribution or clustering analysis must rule out post-fixation aggregation, e.g., by the use of single-binders such as nanobodies. This is in agreement with STED and FRAP data showing that appropriate fixation is critical for imaging of microclusters (49). In contrast, transmembrane proteins with a cytosolic domain such as CD4 or our artificial transmembrane probe are quickly immobilized, indicating an interaction with the submembrane cytoskeleton. During single-particle tracking of membrane probes at 45 Hz, we observed a trend toward faster immobilization in the first few seconds after addition of the pre-warmed chemical fixative. During phase contrast imaging at 0.066 Hz cellular motion was halted within 30 s for both conditions tested. The increased fluctuations in correlation analysis after addition of ice-cold fixative was likely due to thermal effects on sample structure and microscope optics and not diffusion or reaction rate of the fixative. Our observation that CD4 membrane organization is affected by poor actin chemical fixation should serve as a cautionary tale for sample preparation approaches to study membrane proteins. Optimal fixation approaches preserve the cortical actin cytoskeleton structure and the organization of transmembrane proteins in a near-native state (Figure 6). Conversely, suboptimal fixation conditions induce deformations of membrane and cytoskeleton that can result in artifacts that can influence the organization of membrane proteins, such as CD4 (Figure 6). Although, we and others (40, 50–54) suggest that the actin cytoskeleton, protein-protein interactions and the physiological context (e.g., temperature) are important for membrane proteins organization, many studies using SMLM focus on imaging unknown structures and distributions of proteins that do not have a known organization. It is important to highlight that this work does not intend to suggest a direct correlation between the actin cytoskeleton and CD4 surface organization (or other surface proteins). Rather that when performing essential protocol optimization, preservation of the overall cellular structure and physiological context should be a priority. This work also aims to highlight that there are already established protocols that serve as excellent starting points (23, 24, 29, 30), hardware that permits the optimization of such protocols to be streamlined (31, 55) and tools that allow for seamless analysis of possible bottlenecks (22, 55). In conclusion, to extract the most from SMLM experiments it is essential to use reliable and repeatable imaging protocols that preserve, as much as possible, the overall cellular structure.

## Methods

### Cell Lines

COS7 cells were cultured in phenol-red free DMEM (Gibco) supplemented with 2 mM GlutaMAX (Gibco), 50 U/ml penicillin, 50 μg/ml streptomycin (Penstrep, Gibco) and 10% fetal bovine serum (FBS; Gibco). Cells were grown at 37°C in a 5% CO_2_ humidified incubator. Cell lines have not been authenticated.

### Plasmids

The plasmid expressing the calponin homology domain of utrophin fused to GFP (GFP-UtrCH) was a gift from William Bement (33) (Addgene plasmid #26737). The plasmid expressing the cluster of differentiation 4 (CD4) fused to TagRFP-T was constructed for this study by fusing the CD4 (56) and TagRFP-T (57, 58) genes by overlapping PCR, with a 10 amino-acid linker (GGGGSGGGGS) encoded in the overlap primers, and cloning the resulting fragment into pcDNA3.1+ (Thermo Fisher Scientific) using HindIII and XhoI restriction enzymes (Promega). This plasmid is available from Addgene (Addgene plasmid #119238). The plasmid expressing GPI-GFP was a kind gift from Ari Helenius. The plasmid expressing the artificial transmembrane probe was constructed based on Patrick Keller's L-YFP-GT46 (59) by adding the beta-barrel fluorophore mHoneydew on the cytosolic side to increase size (60).

### Live-to-Fixed Super-Resolution Imaging

The NanoJ-Fluidics syringe pump array was installed on a Zeiss Elyra PS.1 microscope equipped with 405, 488, 561, and 642 nm lasers (50, 200, 200, and 160 mW at the optical fiber output). All steps after cell transfection were performed on the microscope, using NanoJ-Fluidics (31, 61). COS7 cells (kind gift from Dr. A. Saiardi) were seeded on ultraclean (62) 25 mm diameter thickness 1.5H coverslips (Marienfeld) at a density of 0.3–0.9 × 105 cells/cm^2^. One day after splitting, cells were transfected with UtrCH-GFP and pCD4-TagRFP-T using Lipofectamine 2000 (Thermo Fisher Scientific) according to the manufacturer's recommendations. Cells were imaged 1–2 days post transfection in culture medium using an Attofluor cell chamber (ThermoFisher), covered with the lid of a 35 mm dish (ThermoFisher), that was kept in place using black non-reflective aluminum tape (T205-1.0 AT205, THORLABs).

Cells were fixed at 4, 23, or 37°C for 15 min with freshly prepared 4% paraformaldehyde (PFA) in the cytoskeleton-preserving buffer “PIPES-EGTA-Magnesium” (PEM: 80 mM PIPES pH 6.8, 5 mM EGTA, 2 mM MgCl_2_) (23) or at 23°C for 15 min with 4% PFA in Phosphate Buffer Saline (PBS: 0.14 M NaCl, 10 mM NaH_2_PO_4_, 10 mM Na_2_HPO_4_).

For stained cells (Figure 2), after fixation cells were permeabilised (PEM with 0.25% Triton-X-100) for 20 min (at 23°C), blocked with blocking buffer [5% Bovine Serum Albumin (BSA) in PEM] for 30 min (at 23°C), and stained with anti-CD4 mAb (OKT4, 6 μg/ml) for 60 min (at 23°C), followed by anti-mouse Alexa Fluor 568 secondary Ab (Molecular Probes) for 60 min (at 23°C).

Structured Illumination Microscopy (SIM) imaging was performed using Plan-Apochromat 63x/1.4 oil DIC M27 objective, in a Zeiss Elyra PS.1 microscope (Zeiss). Images were acquired using 5 phase shifts and 3 grid rotations with the 561 and 488 nm lasers (at 5–10% of maximum output), and filter set 4 (1,851–248, Zeiss). Images were acquired using a sCMOS (pco.edge sCMOS) camera.

Total Internal Reflection Fluorescence (TIRF) imaging of live COS7 cells was performed at 37°C and 5% CO_2_ on a Zeiss Elyra PS.1 microscope with 488 nm and 561 nm laser illumination at 0.5% of maximum output. A 100x TIRF objective (Plan-APOCHROMAT 100x/1.46 Oil, Zeiss) with additional 1.6x magnification was used to collect fluorescence onto an EMCCD camera (iXon Ultra 897, Andor), yielding a pixel size of 100 nm. TIRF STORM imaging of anti-CD4 Alexa Fluor 568 in fixed cells was performed on the same system. 50,000 frames were acquired with 33 ms exposure and 561 nm laser illumination at maximum output power with 405 nm pumping when required (0.5–1% of maximum output when the blinking density was bellow 1 particle/μ m2). STORM imaging was performed in GLOX buffer (150 mM Tris, pH 8, 1% glycerol, 1% glucose, 10 mM NaCl, 1% β-mercaptoethanol, 0.5 mg/ml glucose oxidase, 40 μg/ml catalase). Single-particle tracking was performed in medium at 37°C and 5% CO_2_ on a Zeiss Elyra PS.1 microscope in TIRF mode by acquiring 250/500 frames at 45 FPS with 642 nm laser illumination at 5% of maximum output. Live fixation during phase contrast imaging was performed in medium at 37°C and 5% CO_2_ on a Zeiss Elyra PS.1 microscope at 0.066 FPS with white LED illumination. For live-fixation, medium was replaced by either ice-cold or 37°C pre-warmed 4% PFA in PEM buffer.

### Image Reconstruction and Analysis

For Figure 2 images were processed using the ZEN software (2012, version 8.1.6.484, Zeiss). For channel alignment, a multi-colored bead slide was imaged using the same image acquisition settings. For STORM datasets localizations were detected and rendered using ThunderSTORM (63) with default settings. Fourier Ring Correlation (FRC) values were obtained using NanoJ-SQUIRREL after reconstruction of original data separated into two different stacks composed of odd or even images (22). NanoJ-SQUIRREL and ThunderSTORM are available in Fiji (64). Statistical analysis (ordinary one-way ANOVA) was performed using Prism7 (GraphPad). Single-particle tracking data was analyzed using Trackmate (65) in Fiji and MSDanalyzer (66) in MATLAB (Mathworks). Images sequences for movies were bleach corrected (Fiji) and drift corrected (NanoJ).

Cross-correlation analysis was performed to analyse the stability of samples pre- and post-fixation. Analysis was performed using a custom-written plugin for Fiji (64) using tools from the NanoJ-Core software package (55). Phase contrast images were first drift-corrected using the drift correction functionality of NanoJ-Core. A normalized 2D cross-correlation matrix (CCM) was calculated between each frame of the image series and the frame immediately preceding it. The peak intensity in the CCM indicates the similarity between the two images, where a value of 1.0 indicates perfect similarity between the images. The plugin for this analysis is including in the latest release of the NanoJ-Core software package as “Similarity Evolution”.

## Author Contributions

These contributions follow the contributor roles taxonomy guidelines (https://casrai.org/credit/). PP, DA, CJ, and RH: conceptualization; PP, DA, SC, and CJ: data curation, formal analysis, visualization; PP, DA, and CJ: investigation, methodology, writing original draft; PP, DA, SC, CJ, JM, MM, and RH: resources, validation, writing, review, and editing; PP, DA, JM, MM, and RH: funding acquisition, supervision; PP, DA, and RH: project administration.

### Conflict of Interest Statement

The authors declare that the research was conducted in the absence of any commercial or financial relationships that could be construed as a potential conflict of interest.
