# Supplementary material for: Fix Your Membrane Receptor Imaging: Actin Cytoskeleton and CD4 Membrane Organization Disruption by Chemical Fixation
Source: Front Immunol. 2019 Apr 5;10:675. doi: 10.3389/fimmu.2019.00675 (PMC6460894; doi:10.3389/fimmu.2019.00675)
Supplement: Supplementary file 5 [file Data_Sheet_1.pdf]

Supplementary Material

Supplementary movies corresponding to Figure 5.

Video 1: Time-lapse of vesicle motion during live chemical fixation of COS7 with 4% PFA at 37 °C. Chemical fixative was added at t=0 min. Scale bar 5 µm.

Video 2: Time-lapse of lamellipodia motion during live chemical fixation of COS7 with 4% PFA at 37 °C. Chemical fixative was added at t=0 min. Scale bar 5 µm.

Video 3: Time-lapse of vesicle motion during live chemical fixation of COS7 with 4% PFA at 4 °C. Chemical fixative was added at t=0 min. Scale bar 5 µm.

Video 4: Time-lapse of lamellipodia motion during live chemical fixation of COS7 with 4% PFA at 4 °C. Chemical fixative was added at t=0 min. Scale bar 5 µm.
